# Supplementary material for: Response of bacterial community structure to different ecological niches and their functions in Korean pine forests
Source: PeerJ. 2022 Feb 28;10:e12978. doi: 10.7717/peerj.12978 (PMC8893031; doi:10.7717/peerj.12978)
Supplement: Table S1 [file peerj-10-12978-s001.docx]

**APPENDIX 1**

**Table A1** Information of sample sites

| **Sample Label** | **Northern latitude** | **East Longitude** | **Altitude**  **(m)** | **Vegetation of Korean pine** | **Forest age** | **Sampling time** | **Smples source** |
| --- | --- | --- | --- | --- | --- | --- | --- |
| CS1T1/G1 | 41°56′8″ | 126°30′12″ | 600 | pure artificial forest | 60-year-old | June | rhizospheric soil/root tips |
| CS1T2/G2 | 41°56′8″ | 126°30′12″ | 600 | pure artificial forest | 60-year-old | July | rhizospheric soil/root tips |
| CS1T3/G3 | 41°56′8″ | 126°30′12″ | 600 | pure artificial forest | 60-year-old | August | rhizospheric soil/root tips |
| CS1T4/G4 | 41°56′8″ | 126°30′12″ | 600 | pure artificial forest | 60-year-old | September | rhizospheric soil/root tips |
| CS1T5/G5 | 41°56′8″ | 126°30′12″ | 600 | pure artificial forest | 60-year-old | October | rhizospheric soil/root tips |
| CS1T/G | 41°56′8″ | 126°30′12″ | 600 | pure artificial forest | 60-year-old | August | rhizospheric soil/root tips |
| CS2T/G | 41°59′28″ | 126°37′58″ | 550 | pure artificial forest | 60-year-old | August | rhizospheric soil/root tips |
| CS3T/G | 42°01′45″ | 126°43′30″ | 530 | seedling nursery | 5-year-old | August | rhizospheric soil/root tips |
| CS4T/G | 42°22′55″ | 128°6′1″ | 740 | mixed natural forest | >  150-year-old | August | rhizospheric soil/root tips |
| CS5T/G | 47°11′4″ | 128°52′52″ | 340 | mixed natural forest | >  150-year-old | August | rhizospheric soil/root tips |

**Table A2** Numbers of bacterial OTUs at each classification level identified in different ecological niches

| **Classification** | **entire** | **entire** | **CS1**  **-T1** | **CS1**  **-T2** | **CS1**  **-T3** | **CS1**  **-T4** | **CS1**  **-T5** | **entire** | **CS1**  **-G1** | **CS1**  **-G2** | **CS1**  **-G3** | **CS1**  **-G4** | **CS1**  **-G5** | **entire** | **CS1**  **-T** | **CS2**  **-T** | **CS3**  **-T** | **CS4**  **-T** | **CS5**  **-T** | **entire** | **CS1-G** | **CS2**  **-G** | **CS3**  **-G** | **CS4-G** | **CS5**  **-G** |
| --- | --- | --- | --- | --- | --- | --- | --- | --- | --- | --- | --- | --- | --- | --- | --- | --- | --- | --- | --- | --- | --- | --- | --- | --- | --- |
| **Phylum** | 36 | 35 | 31 | 32 | 33 | 31 | 31 | 25 | 24 | 22 | 17 | 19 | 23 | 35 | 29 | 30 | 31 | 26 | 27 | 27 | 21 | 24 | 19 | 22 | 26 |
| **Class** | 121 | 111 | 107 | 106 | 108 | 109 | 100 | 89 | 54 | 72 | 49 | 53 | 72 | 111 | 105 | 104 | 111 | 83 | 95 | 80 | 62 | 63 | 49 | 56 | 63 |
| **Order** | 221 | 205 | 181 | 177 | 184 | 181 | 186 | 188 | 96 | 135 | 89 | 101 | 115 | 221 | 180 | 183 | 194 | 155 | 165 | 193 | 117 | 108 | 87 | 95 | 114 |
| **Family** | 339 | 305 | 271 | 267 | 273 | 272 | 272 | 243 | 164 | 209 | 144 | 161 | 182 | 321 | 274 | 273 | 288 | 235 | 252 | 257 | 181 | 171 | 142 | 146 | 112 |
| **Genus** | 159 | 137 | 117 | 116 | 112 | 121 | 120 | 127 | 81 | 11 | 79 | 94 | 92 | 151 | 129 | 116 | 131 | 108 | 102 | 135 | 97 | 87 | 90 | 78 | 99 |
| **Species** | 532 | 448 | 382 | 380 | 385 | 384 | 384 | 375 | 253 | 315 | 228 | 254 | 277 | 490 | 384 | 392 | 410 | 345 | 353 | 323 | 276 | 259 | 234 | 225 | 328 |

**Table A3** Related functions predicted of bacteria.

(a) Related functions predicted of bacteria in the rhizospheric soil and in the root tips from June to October (in CS1).

|  | **Functions** | **CS1**  **-G1** | **CS1**  **-G2** | **CS1**  **-G3** | **CS1**  **-G4** | **CS1**  **-G5** | **CS1**  **-T1** | **CS1**  **-T2** | **CS1**  **-T3** | **CS1**  **-T4** | **CS1**  **-T5** |
| --- | --- | --- | --- | --- | --- | --- | --- | --- | --- | --- | --- |
| Nitrogen cycle | aerobic ammonia oxidation | 0 | 0 | 0 | 0 | 0 | 10 | 4 | 9 | 5 | 6 |
|  | aerobic nitrite oxidation | 7 | 16 | 11 | 24 | 14 | 558 | 534 | 454 | 426 | 547 |
|  | nitrification | 7 | 16 | 11 | 24 | 14 | 568 | 538 | 463 | 431 | 553 |
|  | nitrate denitrification | 65 | 276 | 88 | 85 | 95 | 612 | 511 | 479 | 482 | 466 |
|  | nitrite denitrification | 65 | 276 | 88 | 85 | 95 | 612 | 511 | 479 | 482 | 466 |
|  | nitrous oxide denitrification | 65 | 276 | 88 | 85 | 95 | 612 | 511 | 479 | 482 | 466 |
|  | nitrogen fixation | 113 | 623 | 184 | 182 | 274 | 271 | 255 | 146 | 325 | 279 |
|  | nitrate ammonification | 745 | 1106 | 1817 | 1047 | 136 | 0 | 1 | 0 | 0 | 2 |
|  | nitrite ammonification | 745 | 1107 | 1818 | 1047 | 136 | 0 | 1 | 0 | 0 | 2 |
|  | nitrite respiration | 810 | 1383 | 1906 | 1132 | 231 | 612 | 512 | 479 | 482 | 468 |
|  | nitrate respiration | 818 | 1412 | 1922 | 1143 | 263 | 625 | 524 | 495 | 486 | 476 |
|  | nitrate reduction | 4354 | 4420 | 5321 | 8915 | 2236 | 676 | 549 | 516 | 531 | 565 |
|  | nitrogen respiration | 818 | 1412 | 1922 | 1143 | 263 | 625 | 524 | 495 | 486 | 476 |
| phototrophy | anoxygenic photoautotrophy sulfur oxidizing | 65 | 276 | 88 | 85 | 95 | 612 | 511 | 479 | 482 | 466 |
|  | anoxygenic photoautotrophy | 69 | 333 | 119 | 130 | 130 | 629 | 526 | 498 | 507 | 489 |
|  | photoautotrophy | 70 | 337 | 119 | 132 | 131 | 639 | 533 | 511 | 521 | 501 |
|  | photoheterotrophy | 73 | 344 | 120 | 135 | 132 | 637 | 532 | 506 | 521 | 496 |
|  | phototrophy | 74 | 348 | 120 | 137 | 133 | 647 | 539 | 519 | 535 | 508 |
| chemoheterotrophy | chemoheterotrophy | 19933 | 14547 | 21420 | 27939 | 20121 | 2391 | 2041 | 1756 | 2255 | 2797 |
|  | aerobic chemoheterotrophy | 15725 | 10403 | 16206 | 19167 | 17997 | 2320 | 1979 | 1713 | 2183 | 2683 |

(b) Related functions predicted of bacteria in the rhizospheric soil and in the root tips in different forests with age (in CS1, CS2, CS3, CS4 and CS5).

|  | **Functions** | **CS1-G** | **CS2-G** | **CS3-G** | **CS4-G** | **CS5-G** | **CS1-T** | **CS2-T** | **CS3-T** | **CS4-T** | **CS5-T** |
| --- | --- | --- | --- | --- | --- | --- | --- | --- | --- | --- | --- |
| Nitrogen cycle | aerobic ammonia oxidation | 0 | 0 | 0 | 0 | 0 | 3 | 1 | 17 | 2 | 4 |
|  | aerobic nitrite oxidation | 20 | 13 | 5 | 4 | 68 | 518 | 491 | 390 | 184 | 425 |
|  | nitrification | 20 | 13 | 5 | 4 | 68 | 521 | 492 | 407 | 186 | 429 |
|  | nitrate denitrification | 106 | 135 | 38 | 96 | 1077 | 516 | 533 | 346 | 890 | 1608 |
|  | nitrite denitrification | 106 | 135 | 38 | 96 | 1077 | 516 | 533 | 346 | 890 | 1608 |
|  | nitrous oxide denitrification | 106 | 135 | 38 | 96 | 1077 | 516 | 533 | 346 | 890 | 1608 |
|  | nitrogen fixation | 217 | 480 | 189 | 377 | 2028 | 202 | 172 | 81 | 543 | 413 |
|  | nitrate ammonification | 681 | 1152 | 372 | 1865 | 612 | 3 | 6 | 0 | 138 | 0 |
|  | nitrite ammonification | 683 | 1152 | 372 | 1865 | 616 | 3 | 6 | 1 | 138 | 0 |
|  | nitrite respiration | 789 | 1287 | 410 | 1961 | 1693 | 519 | 539 | 347 | 1028 | 1608 |
|  | nitrate respiration | 804 | 1326 | 418 | 1976 | 1811 | 529 | 552 | 361 | 1043 | 1679 |
|  | nitratereduction | 2857 | 5002 | 4787 | 7968 | 5510 | 570 | 940 | 482 | 1985 | 1696 |
|  | nitrogenrespiration | 804 | 1326 | 418 | 1976 | 1811 | 529 | 552 | 361 | 1043 | 1679 |
| phototrophy | anoxygenic photoautotrophy sulfur oxidizing | 106 | 135 | 38 | 96 | 1077 | 516 | 533 | 346 | 890 | 1608 |
|  | anoxygenic photoautotrophy | 125 | 203 | 118 | 133 | 1337 | 523 | 551 | 379 | 935 | 1617 |
|  | photoautotrophy | 129 | 204 | 119 | 134 | 1337 | 537 | 557 | 413 | 939 | 1631 |
|  | photoheterotrophy | 132 | 210 | 200 | 133 | 1342 | 535 | 558 | 405 | 936 | 1620 |
|  | phototrophy | 136 | 211 | 201 | 134 | 1342 | 549 | 564 | 439 | 940 | 1634 |
| chemoheterotrophy | chemoheterotrophy | 18292 | 18075 | 19310 | 17801 | 13778 | 2037 | 3093 | 1976 | 5573 | 3839 |
|  | aerobic chemoheterotrophy | 15605 | 13396 | 14514 | 9922 | 9257 | 1977 | 2911 | 1897 | 4471 | 3693 |

**Table A4** Spearman correlation analysis biomaker of the root tips with other bacterial taxa in the root tips and its rhizospheric soil.

(a) Bacteria biomaker families in the root tips with Bacteria families in its rhizospheric soil.

| **Bacteria biomaker**  **families**  **in the root tips** | **Bacteria families**  **in the**  **rhizospheric soil** | Spearman correlation coefficient (r) | ***p*** |
| --- | --- | --- | --- |
| Enterobacteriaceae | oc28 | -0.950 | 0.007 |
| Moraxellaceae (bb) | Gemmataceae | 0.952 | 0.006 |
|  | Sphingomonadaceae | 0.943 | 0.008 |
| Pseudomonadaceae (bb) | Nocardioidaceae | -0.949 | 0.007 |
| Rhizobiaceae (bb) | Xanthomonadaceae | -0.943 | 0.000 |
| Burkholderiaceae (bb) | Bacillaceae | 0.949 | 0.000 |
|  | Frankiaceae | 0.938 | 0.000 |

(b) Bacteria biomaker families with other bacterial families in the root tips.

| **Bacteria biomaker families**  **in the root tips** | **Other bacterial families**  **in the root tips** | **Spearman correlation coefficient (r)** | ***p*** |
| --- | --- | --- | --- |
| Enterobacteriaceae | Sphingobacteriaceae | -0.969 | 0.003 |
| Moraxellaceae | Comamonadaceae | -0.954 | 0.006 |
| Pseudomonadaceae | FFCH4570 | -0.959 | 0.005 |
| Rhizobiaceae | Oxalobacteraceae | -0.954 | 0.000 |
| Burkholderiaceae | Oxalobacteraceae | -0.956 | 0.000 |
