# Supplementary material for: Response of bacterial community structure to different ecological niches and their functions in Korean pine forests
Source: PeerJ. 2022 Feb 28;10:e12978. doi: 10.7717/peerj.12978 (PMC8893031; doi:10.7717/peerj.12978)
Supplement: Table S2 [file peerj-10-12978-s002.docx]

**Supplementary Table S2. Numbers of bacterial OTUs at each classification level identified in different ecological niches**

| **Classification** | **entire** | **entire** | **CS1**  **-T1** | **CS1**  **-T2** | **CS1**  **-T3** | **CS1**  **-T4** | **CS1**  **-T5** | **entire** | **CS1**  **-G1** | **CS1**  **-G2** | **CS1**  **-G3** | **CS1**  **-G4** | **CS1**  **-G5** | **entire** | **CS1**  **-T** | **CS2**  **-T** | **CS3**  **-T** | **CS4**  **-T** | **CS5**  **-T** | **entire** | **CS1-G** | **CS2**  **-G** | **CS3**  **-G** | **CS4-G** | **CS5**  **-G** |
| --- | --- | --- | --- | --- | --- | --- | --- | --- | --- | --- | --- | --- | --- | --- | --- | --- | --- | --- | --- | --- | --- | --- | --- | --- | --- |
| **Phylum** | 36 | 35 | 31 | 32 | 33 | 31 | 31 | 25 | 24 | 22 | 17 | 19 | 23 | 35 | 29 | 30 | 31 | 26 | 27 | 27 | 21 | 24 | 19 | 22 | 26 |
| **Class** | 121 | 111 | 107 | 106 | 108 | 109 | 100 | 89 | 54 | 72 | 49 | 53 | 72 | 111 | 105 | 104 | 111 | 83 | 95 | 80 | 62 | 63 | 49 | 56 | 63 |
| **Order** | 221 | 205 | 181 | 177 | 184 | 181 | 186 | 188 | 96 | 135 | 89 | 101 | 115 | 221 | 180 | 183 | 194 | 155 | 165 | 193 | 117 | 108 | 87 | 95 | 114 |
| **Family** | 339 | 305 | 271 | 267 | 273 | 272 | 272 | 243 | 164 | 209 | 144 | 161 | 182 | 321 | 274 | 273 | 288 | 235 | 252 | 257 | 181 | 171 | 142 | 146 | 112 |
| **Genus** | 159 | 137 | 117 | 116 | 112 | 121 | 120 | 127 | 81 | 11 | 79 | 94 | 92 | 151 | 129 | 116 | 131 | 108 | 102 | 135 | 97 | 87 | 90 | 78 | 99 |
| **Species** | 532 | 448 | 382 | 380 | 385 | 384 | 384 | 375 | 253 | 315 | 228 | 254 | 277 | 490 | 384 | 392 | 410 | 345 | 353 | 323 | 276 | 259 | 234 | 225 | 328 |
