# Supplementary material for: Response of bacterial community structure to different ecological niches and their functions in Korean pine forests
Source: PeerJ. 2022 Feb 28;10:e12978. doi: 10.7717/peerj.12978 (PMC8893031; doi:10.7717/peerj.12978)
Supplement: Table S3 [file peerj-10-12978-s003.docx]

**Supplementary Table S4 Spearman correlation analysis biomaker of the root tips with other bacterial taxa in the root tips and its rhizospheric soil.**

(a) Bacteria biomaker families in the root tips with Bacteria families in its rhizospheric soil.

| **Bacteria biomaker**  **families**  **in the root tips** | **Bacteria families**  **in the**  **rhizospheric soil** | Spearman correlation coefficient (r) | ***p*** |
| --- | --- | --- | --- |
| Enterobacteriaceae | oc28 | -0.950 | 0.007 |
| Moraxellaceae (bb) | Gemmataceae | 0.952 | 0.006 |
|  | Sphingomonadaceae | 0.943 | 0.008 |
| Pseudomonadaceae (bb) | Nocardioidaceae | -0.949 | 0.007 |
| Rhizobiaceae (bb) | Xanthomonadaceae | -0.943 | 0.000 |
| Burkholderiaceae (bb) | Bacillaceae | 0.949 | 0.000 |
|  | Frankiaceae | 0.938 | 0.000 |

(b) Bacteria biomaker families with other bacterial families in the root tips.

| **Bacteria biomaker families**  **in the root tips** | **Other bacterial families**  **in the root tips** | **Spearman correlation coefficient (r)** | ***p*** |
| --- | --- | --- | --- |
| Enterobacteriaceae | Sphingobacteriaceae | -0.969 | 0.003 |
| Moraxellaceae | Comamonadaceae | -0.954 | 0.006 |
| Pseudomonadaceae | FFCH4570 | -0.959 | 0.005 |
| Rhizobiaceae | Oxalobacteraceae | -0.954 | 0.000 |
| Burkholderiaceae | Oxalobacteraceae | -0.956 | 0.000 |
