# Supplementary material for: Response of bacterial community structure to different ecological niches and their functions in Korean pine forests
Source: PeerJ. 2022 Feb 28;10:e12978. doi: 10.7717/peerj.12978 (PMC8893031; doi:10.7717/peerj.12978)
Supplement: Data S1 [file peerj-10-12978-s004.docx]

**Supplementary Table 4 Soil physical and chemical properties.**

| **Samples** | **pH** | **SAN**  **(mg·kg^-1^)** | **SAK**  **(mg·kg^-1^)** | **SAP**  **(mg·kg^-1^)** | **SOM**  **(g·kg^-1^)** | MT  (ºC) | MR  (mm) |
| --- | --- | --- | --- | --- | --- | --- | --- |
| CS1-June1 | 6.2101 | 190.1090 | 297.4294 | 2.2762 | 113.8471 | 17.4 | 74.8 |
| CS1-June2 | 6.3423 | 176.6770 | 287.6177 | 1.9727 | 116.9828 |  |  |
| CS1-June3 | 6.3310 | 180.8100 | 302.3352 | 2.1203 | 114.8244 |  |  |
| CS1-July1 | 6.5301 | 195.2750 | 253.2771 | 1.6692 | 70.0772 | 22.7 | 172.2 |
| CS1-July2 | 6.4823 | 225.2380 | 263.0887 | 2.8832 | 69.7919 |  |  |
| CS1-July3 | 6.4723 | 229.3700 | 263.0887 | 2.3002 | 67.4902 |  |  |
| CS1-August1 | 6.4523 | 270.6980 | 253.2771 | 1.0905 | 99.2576 | 23.9 | 317.0 |
| CS1-August2 | 6.3495 | 215.9390 | 248.3713 | 1.5175 | 99.7332 |  |  |
| CS1-August3 | 6.2934 | 256.2340 | 253.2771 | 1.0301 | 104.9302 |  |  |
| CS1-September1 | 6.3833 | 216.9720 | 253.2771 | 4.7041 | 132.1464 | 15.0 | 78.6 |
| CS1-September2 | 6.2754 | 220.0720 | 258.1829 | 2.6119 | 133.0648 |  |  |
| CS1-September3 | 6.2243 | 216.9720 | 272.9003 | 3.4033 | 136.9412 |  |  |
| CS1-October1 | 6.2365 | 213.8720 | 263.0887 | 2.4279 | 114.2845 | 7.8 | 70.2 |
| CS1-October2 | 6.4334 | 209.7400 | 253.2771 | 0.9470 | 116.9094 |  |  |
| CS1-October3 | 6.2198 | 220.0720 | 272.9003 | 1.1980 | 105.0058 |  |  |
| CS1-1 | 6.4523 | 270.6980 | 253.2771 | 1.0905 | 99.2576 | 23.9 | 317.00 |
| CS1-2 | 6.3495 | 215.9390 | 248.3713 | 1.5175 | 99.7332 |  |  |
| CS1-3 | 6.2934 | 256.2340 | 253.2771 | 1.0301 | 104.9302 |  |  |
| CS2-1 | 6.1876 | 233.5030 | 272.9003 | 7.5417 | 114.9012 | 21.40 | 373.80 |
| CS2-2 | 6.0956 | 225.2380 | 282.7119 | 7.7798 | 121.1366 |  |  |
| CS2-3 | 6.1342 | 231.4370 | 287.6177 | 7.9970 | 118.3350 |  |  |
| CS3-1 | 6.5865 | 227.3040 | 189.5016 | 12.5948 | 141.1269 | 21.30 | 336.90 |
| CS3-2 | 6.8994 | 236.6030 | 179.6900 | 4.7856 | 145.0893 |  |  |
| CS3-3 | 7.1578 | 231.4370 | 174.7841 | 8.1341 | 129.0492 |  |  |
| CS4-1 | 5.2795 | 385.3840 | 498.5675 | 5.1503 | 230.2212 | 19.60 | 297.40 |
| CS4-2 | 5.3368 | 364.7200 | 503.4733 | 8.8012 | 235.5923 |  |  |
| CS4-3 | 5.5942 | 368.8520 | 493.6617 | 7.0031 | 225.7769 |  |  |
| CS5-1 | 5.3649 | 326.4910 | 302.3352 | 9.4582 | 181.1808 | 18.40 | 186.00 |
| CS5-2 | 5.7478 | 321.3250 | 307.2410 | 6.6768 | 188.1862 |  |  |
| CS5-3 | 5.8674 | 317.1920 | 302.3352 | 7.9862 | 186.8139 |  |  |
